# Trajectory of Viral RNA Load Among Persons With Incident SARS-CoV-2 G614 Infection (Wuhan Strain) in Association With COVID-19 Symptom Onset and Severity

**DOI:** 10.1001/jamanetworkopen.2021.42796

**Published:** 2022-01-10

**Authors:** Helen C. Stankiewicz Karita, Tracy Q. Dong, Christine Johnston, Kathleen M. Neuzil, Michael K. Paasche-Orlow, Patricia J. Kissinger, Anna Bershteyn, Lorna E. Thorpe, Meagan Deming, Angelica Kottkamp, Miriam Laufer, Raphael J. Landovitz, Alfred Luk, Risa Hoffman, Pavitra Roychoudhury, Craig A. Magaret, Alexander L. Greninger, Meei-Li Huang, Keith R. Jerome, Mark Wener, Connie Celum, Helen Y. Chu, Jared M. Baeten, Anna Wald, Ruanne V. Barnabas, Elizabeth R. Brown

**Affiliations:** 1 Division of Allergy and Infectious Diseases, University of Washington, Seattle; 2 Vaccine and Infectious Disease Division, Fred Hutchinson Cancer Research Center, Seattle, Washington; 3 Department of Laboratory Medicine and Pathology, University of Washington, Seattle; 4 Department of Medicine, University of Maryland School of Medicine, Baltimore; 5 Department of Medicine, Boston University School of Medicine, Boston, Massachusetts; 6 Department of Medicine, Boston Medical Center, Boston, Massachusetts; 7 Department of Epidemiology, Tulane University, New Orleans, Louisiana; 8 Department of Population Health, New York University Grossman School of Medicine, New York; 9 Department of Medicine, New York University Grossman School of Medicine, New York; 10 Department of Epidemiology and Public Health, University of Maryland School of Medicine, Baltimore; 11 Department of Medicine, University of California, Los Angeles, Los Angeles; 12 Department of Medicine, Tulane University, New Orleans, Louisiana; 13 Division of Rheumatology, University of Washington, Seattle; 14 Department of Global Health, University of Washington, Seattle; 15 Department of Epidemiology, University of Washington, Seattle; 16 Department of Biostatistics, University of Washington, Seattle; 17 Public Health Sciences Division, Fred Hutchinson Cancer Research Center, Seattle, Washington

## Abstract

**Importance:**

The SARS-CoV-2 viral trajectory has not been well characterized in incident infections. These data are needed to inform natural history, prevention practices, and therapeutic development.

**Objective:**

To characterize early SARS-CoV-2 viral RNA load (hereafter referred to as viral load) in individuals with incident infections in association with COVID-19 symptom onset and severity.

**Design, Setting, and Participants:**

This prospective cohort study was a secondary data analysis of a remotely conducted study that enrolled 829 asymptomatic community-based participants recently exposed (<96 hours) to persons with SARS-CoV-2 from 41 US states from March 31 to August 21, 2020. Two cohorts were studied: (1) participants who were SARS-CoV-2 negative at baseline and tested positive during study follow-up, and (2) participants who had 2 or more positive swabs during follow-up, regardless of the initial (baseline) swab result. Participants collected daily midturbinate swab samples for SARS-CoV-2 RNA detection and maintained symptom diaries for 14 days.

**Exposure:**

Laboratory-confirmed SARS-CoV-2 infection.

**Main Outcomes and Measures:**

The observed SARS-CoV-2 viral load among incident infections was summarized, and piecewise linear mixed-effects models were used to estimate the characteristics of viral trajectories in association with COVID-19 symptom onset and severity.

**Results:**

A total of 97 participants (55 women [57%]; median age, 37 years [IQR, 27-52 years]) developed incident infections during follow-up. Forty-two participants (43%) had viral shedding for 1 day (median peak viral load cycle threshold [Ct] value, 38.5 [95% CI, 38.3-39.0]), 18 (19%) for 2 to 6 days (median Ct value, 36.7 [95% CI, 30.2-38.1]), and 31 (32%) for 7 days or more (median Ct value, 18.3 [95% CI, 17.4-22.0]). The cycle threshold value has an inverse association with viral load. Six participants (6%) had 1 to 6 days of viral shedding with censored duration. The peak mean (SD) viral load was observed on day 3 of shedding (Ct value, 33.8 [95% CI, 31.9-35.6]). Based on the statistical models fitted to 129 participants (60 men [47%]; median age, 38 years [IQR, 25-54 years]) with 2 or more SARS-CoV-2–positive swab samples, persons reporting moderate or severe symptoms tended to have a higher peak mean viral load than those who were asymptomatic (Ct value, 23.3 [95% CI, 22.6-24.0] vs 30.7 [95% CI, 29.8-31.4]). Mild symptoms generally started within 1 day of peak viral load, and moderate or severe symptoms 2 days after peak viral load. All 535 sequenced samples detected the G614 variant (Wuhan strain).

**Conclusions and Relevance:**

This cohort study suggests that having incident SARS-CoV-2 G614 infection was associated with a rapid viral load peak followed by slower decay. COVID-19 symptom onset generally coincided with peak viral load, which correlated positively with symptom severity. This longitudinal evaluation of the SARS-CoV-2 G614 with frequent molecular testing serves as a reference for comparing emergent viral lineages to inform clinical trial designs and public health strategies to contain the spread of the virus.

## Key Points

**Question:**

What are the characteristics of SARS-CoV-2 G614 viral shedding in incident infections in association with COVID-19 symptom onset and severity?

**Findings:**

In a cohort study of persons who tested positive for SARS-CoV-2 after recent exposure, viral RNA trajectory was characterized by a rapid peak followed by slower decay. Peak viral load correlated positively with symptom severity and generally occurred within 1 day of symptom onset if the patient was symptomatic.

**Meaning:**

A detailed description of the SARS-CoV-2 G614 viral shedding trajectory serves as a baseline for comparison with new viral variants of concern and informs models to plan clinical trials to end the pandemic.

## Teaser

This cohort study characterizes early SARS-CoV-2 viral RNA load in individuals with incident infections in association with COVID-19 symptom onset and severity.

## Introduction

Since December 2019, COVID-19 has caused more than 5 million deaths worldwide.^1^ Despite the substantial advancement in public health prevention approaches and the deployment of vaccines, the pandemic continues because less than 50% of the global population has received any vaccine doses.^2^ Furthermore, the emergence and spread of novel variants of concern have compelled global attention because of increased transmissibility and potential virulence.^3,4,5,6,7^ Novel variants, including the Delta variant, have outcompeted the wild-type virus and the earlier-identified variants as the dominant circulating strain in the US and many other countries.^3,5,8,9^

Substantial uncertainty remains regarding the natural history of SARS-CoV-2 and early viral shedding. Studies of frequently monitored hospitalized patients with COVID-19 have provided data on the viral RNA load among persions with SARS-CoV-2 from diagnosis through the course of the infection.^10,11,12,13^ Among outpatients, very few prospective studies have captured the dynamics and symptoms of the virus from the acquisition of infection rather than at the time of diagnosis.^14,15^ As the continued spread of SARS-CoV-2 allows for the appearance of new viral and potentially deleterious variants, the characterization of early viral shedding trajectories is essential to inform the likelihood of transmission and to improve models of transmission dynamics to optimize appropriate infection control interventions. With the threat of emerging resistance to passive immunity from monoclonal antibodies and acquired immunity generated by current COVID-19 vaccines, frequent viral load measurements in clinical trials evaluating new therapeutics are crucial to measure objective disease markers and to allow for an accurate assessment of the effect of therapy.

Prior to the documented emergence of new SARS-CoV-2 variants of concern, we enrolled asymptomatic persons exposed to SARS-CoV-2 in a prospective study. Within a median of 1 to 2 days after exposure to household, patient, or social contacts with virologically documented SARS-CoV-2 infection, participants self-collected midturbinate swab samples daily for 14 days to obtain SARS-CoV-2 RNA cycle threshold (Ct) values via real-time reverse transcription–polymerase chain reaction (RT-PCR) assay, along with daily symptom surveys. We examined the observed SARS-CoV-2 viral RNA load (hereafter referred to as viral load) in participants with a negative swab sample at baseline and used statistical models to estimate the timing and magnitude of the SARS-CoV-2 viral trajectory in association with COVID-19 symptom onset and severity in participants with 2 or more positive RT-PCR test results. The results provide detailed SARS-CoV-2 G614 strain trajectories to establish a baseline for comparison with new viral variants detected in genomic surveillance programs.

## Methods

### Study Population and Procedures

We analyzed data from the COVID-19 PEP (Postexposure Prophylaxis) Study, a double-blind, household-randomized clinical trial comparing hydroxychloroquine with a placebo-like control for prevention of SARS-CoV-2 infection.^16^ The study was remotely conducted between March 31 and August 21, 2020, and enrolled 829 asymptomatic close contacts (defined as a household contact residing in the same residence of an index case, a household contact with prolonged exposure to confined spaces, and/or a health care worker who cared for the index case without appropriate personal protective equipment) recently exposed (<96 hours) to persons with laboratory-confirmed SARS-CoV-2 infection from 41 US states. The study found no differences between the hydroxychloroquine and placebo-like control groups in SARS-CoV-2 acquisition, and no participants received COVID-19 vaccines. As such, we examined the natural history of SARS-CoV-2 among participants regardless of assigned group. For the present analyses, we evaluated 2 participant cohorts: (1) participants who had a negative SARS-CoV-2 swab sample at baseline and acquired SARS-CoV-2 during the study follow-up and (2) participants who had 2 or more positive swab samples during follow-up (eFigure 1 in the Supplement). The Western Institutional Review Board approved the trial; all participants provided written informed consent. This study followed the Strengthening the Reporting of Observational Studies in Epidemiology (STROBE) reporting guideline and was not an a priori planned analysis of the parent trial.

Participants self-collected daily midturbinate swab samples, which were shipped to the University of Washington every 5 to 7 days,^16^ for 14 consecutive days. Participants completed daily symptom questionnaires using REDCap.^17^ COVID-19 symptoms and severity were evaluated using the Centers for Disease Control and Prevention clinical case definition criteria and a symptom severity scale (eTable 1 in the Supplement).^18^

### Laboratory Methods

Swab samples were tested for SARS-CoV-2 RNA at the University of Washington Virology Laboratory using RT-PCR targeting regions N1 and N2 of the nucleocapsid gene.^19^ To document adequate swab technique as indicated by the presence of cellular DNA, a subset of swab samples were tested for human DNA marker ribonuclease P (1207 swab samples from 288 participants; 11% of all collected swab samples). Whole-genome sequencing was attempted on all samples with a Ct value of less than 34. Sequencing libraries were prepared using Swift Biosciences’ Normalase amplicon panel and sequenced on Illumina Nextseq instruments using 2 × 150 reads.^20^

### Statistical Analysis

Analyses were conducted on 2 cohorts of participants. The incident infection cohort consisted of participants who did not have SARS-CoV-2 RNA detected at baseline but later had detectable RNA and thus could potentially have their entire viral load trajectory observed. Analyses based on this cohort rely only on summaries of the observed data. The trajectory modeling cohort was defined to include all participants with 2 or more swab samples with detectable SARS-CoV-2 RNA in the first 14 days of follow-up, regardless of baseline status, to maximize use of data from participants with sustained viral detection. Because the viral load trajectories for some of these participants were left truncated, analysis of this cohort was model based.

### Viral Shedding Characteristics in Incident Infections

This subsection describes analyses conducted on the incident infection cohort. The duration of viral shedding was defined as the number of days from the first positive swab sample until the last positive swab sample. Censoring was indicated if a participant had a positive swab sample on the last day of swab sample collection. For a given day, a participant’s viral load was calculated as the mean of N1 and N2 Ct values from the RT-PCR. The Ct value assigned to the negative samples was 40. The cohort was divided into 4 shedding duration subgroups based on interval testing in prior longitudinal studies of viral trajectories^21,22^ (1 day, 2-6 days, ≥7 days, and censored with 1-6 days of observed shedding). Participants reached the observed peak viral load when the sample yielded the minimum Ct value, indicating the highest quantity of virus detected. If this peak occurred on the last day of swabbing, it was treated as censored (the peak could have been this value or higher). Because we have right-censored data, the Kaplan-Meier method was used to calculate the median peak viral load in Ct values and the associated 95% CIs within each shedding duration subgroup, accounting for clustering at the household level.^23^ The individual observed peak viral load in Ct values and the median values were plotted within each shedding duration subgroup.

The mean SARS-CoV-2 viral load by day of viral shedding was plotted among all participants and within those who did or did not report COVID-19 symptoms during follow-up. The time and magnitude of the mean peak viral load were reported and compared across the groups.

### Model-Based Viral Shedding Characteristics in Association With COVID-19 Symptom Onset and Severity

We conducted model-based analyses on the trajectory modeling cohort. We grouped participants by reported symptoms: (1) asymptomatic, defined as no COVID-19 symptoms throughout follow-up; (2) mildly symptomatic, defined as reporting only mild COVID-19 symptoms during follow-up; and (3) moderately or severely symptomatic, defined as reporting at least 1 day of moderate or severe COVID-19 symptoms during follow-up. We developed piecewise linear mixed-effects models to estimate the viral load trajectories in Ct values for every person within each group (eMethods and eFigure 2 in the Supplement). We compared 3 viral dynamics characteristics across symptom groups: magnitude of peak viral load, time from viral shedding onset to peak, and time from peak to shedding cessation. We estimated and compared the time from peak viral load to onset of mild or moderate symptoms for people in the symptomatic groups. Analyses were performed using R, version 4.1^24^ and JAGS, version 4.3.^25^

## Results

### Study Participants

Of the 829 participants in the parent study, 97 (12%; 55 women [57%]; median age, 37 years [IQR, 27-52 years]) had a negative SARS-CoV-2 swab sample at baseline and at least 1 subsequent positive swab sampple, and 129 (16%; 60 men [47%]; median age, 38 years [IQR, 25-54 years]) had at least 2 positive swab samples during the 14 days of follow-up (eTable 2 in the Supplement). More than 90% of both cohorts (incident infection cohort, 90 of 97 [93%]; and trajectory modeling cohort, 119 of 129 [92%]) provided 14 swab samples, and more than 80% (incident infection cohort, 82 of 97 [85%]; and trajectory modeling cohort, 105 of 129 [81%]) completed all daily symptom surveys. The median time from last exposure to index case(s) to first swab sample collection was 43 hours (IQR, 21-74 hours) and 42 hours (IQR, 12-72 hours) for each cohort, respectively. Ribonuclease P was detected in 99% (1197 of 1207) of samples tested (eFigure 3 in the Supplement). A total of 535 samples with a Ct value of less than 34, obtained from 108 participants, were sequenced. Sequenced genomes were assigned to 30 lineages that belonged to the G614 variant (ie, the Wuhan strain; eTable 3 in the Supplement).

### SARS-CoV-2 Characteristics in Incident Infections

**Figure 1.**
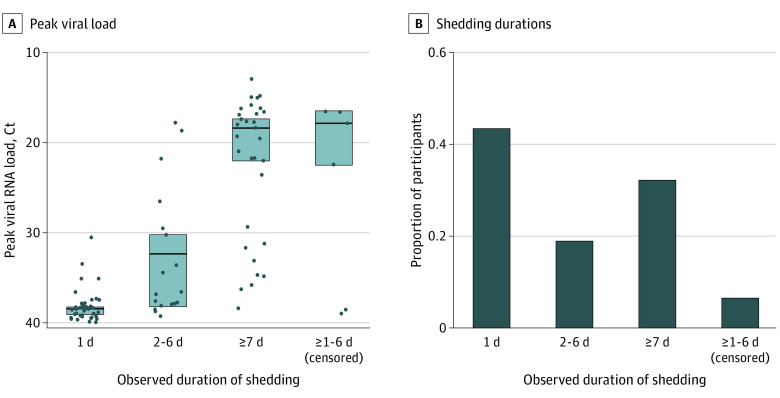
Observed Shedding Duration and Peak Viral Load Among 97 Participants With Incident SARS-CoV-2 Infection (Incident Infection Cohort) We grouped the participants according to the observed duration of shedding in days. Each dot indicates a participant’s observed peak viral RNA load, defined as the minimum mean cycle threshold (Ct) value measured during study follow-up. The horizontal line segments indicate the median peak viral load in each shedding duration group, and the shaded areas indicate the 95% CIs around the median values, calculated using the Kaplan-Meier method that accounted for censoring and clustering within households.

Individual viral load trajectories for the 97 persons with incident SARS-CoV-2 infection are presented in eFigure 4 in the Supplement. Of 97 participants, 42 (43%) had viral shedding detected for 1 day, 18 (19%) for 2 to 6 days, and 31 (32%) for 7 days or more (Figure 1). Six persons (6%) had 1 to 6 days of observed shedding but were right-censored. A lower median peak Ct value (indicating higher levels of viral RNA) was observed in participants with longer duration of shedding. The median peak viral load measured in Ct values was 38.5 (95% CI, 38.3-39.0) in persons with viral shedding for 1 day, 36.7 (95% CI, 30.2-38.1) for those with viral shedding for 2 to 6 days, and 18.3 (95% CI, 17.4-22.0) for those with viral shedding for 7 days or more (Figure 1).

**Figure 2.**
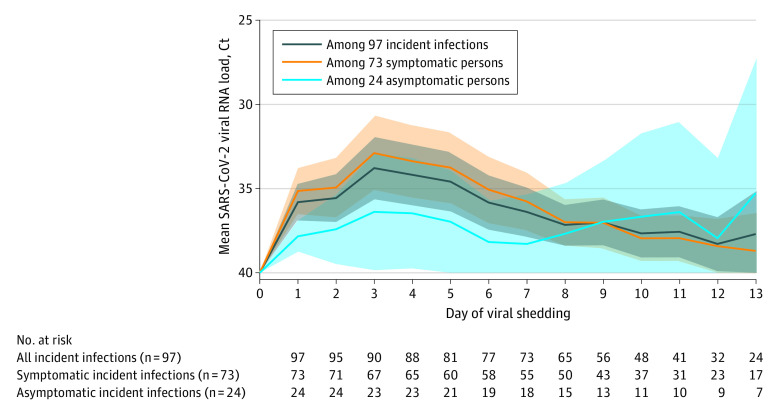
Mean SARS-CoV-2 Viral RNA Load Cycle Threshold (Ct) Values by Day of Viral Shedding During the 14-Day Study Follow-up The shaded areas indicate the pointwise 95% CIs of the mean viral RNA load. The sample sizes for the mean viral load calculations are presented in the table. Centers for Disease Control and Prevention clinical criteria were used to ascertain symptomatic COVID-19 cases. Fewer than 16 asymptomatic persons were used to calculate the mean viral load after day 7 of viral shedding.

In 73 persons who reported COVID-19 symptoms, the peak mean (SD) viral load was observed on day 3 of viral shedding (Ct value, 32.9 [95% CI, 30.7-35.1]) (Figure 2). Among 24 asymptomatic persons, the mean (SD) viral load peaked on day 3 of viral shedding (Ct value, 36.4 [95% CI, 32.9-39.8]), then decreased before increasing again after day 7. The sample sizes for the mean viral load calculations are presented in Figure 2.

### Model-Based Viral Shedding Characteristics in Association With COVID-19 Symptom Onset and Severity

**Figure 3.**
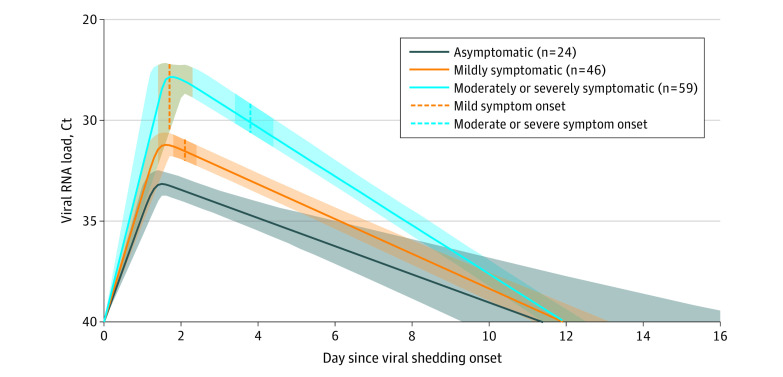
Model-Estimated Population-Level Viral RNA Load Trajectories in Cycle Threshold (Ct) Values for Participants Infected With SARS-CoV-2 Who Had at Least 2 Positive Swab Samples During Study Follow-up The estimated time from onset of shedding to presentation of mild symptoms or moderate or severe symptoms is indicated by the vertical dashed lines, with the shaded areas indicating 95% credible intervals of the associated curve and its bounds.

Of 129 participants in the trajectory modeling cohort, 24 (19%) were asymptomatic, 46 (36%) reported mild COVID-19 symptoms, and 59 (46%) reported moderate or severe symptoms (Table; eFigure 5 in the Supplement). The estimated mean peak viral load was the lowest in the asymptomatic group (Ct value, 30.7; 95% CI, 29.8-31.4), followed by the mildly symptomatic group (Ct value, 28.0; 95% CI, 27.3-28.5) and the moderately or severely symptomatic group (Ct value, 23.3; 95% CI, 22.6-24.0) (Table). Time from viral shedding onset to peak viral load correlated positively with symptom severity: 1.3 days (95% CI, 1.0-1.6 days) for the asymptomatic group, 1.4 days (95% CI, 1.2-1.9 days) for the mildly symptomatic group, and 1.7 days (95% CI, 1.2-2.2 days) for the moderately or severely symptomatic group. After reaching the peak viral load, shedding cessation occurred at similar times in the 3 groups: 10.4 days (95% CI, 8.0-15.9 days) for the asymptomatic group, 10.5 days (95% CI, 9.8-11.6 days) for the mildly symptomatic group, and 10.3 days (95% CI, 9.9-11.0 days) for the moderately or severely symptomatic group (Table; Figure 3; eFigure 6 in the Supplement).

**Table.** Data on Viral RNA Load Trajectory and Symptom Onset Relative to Onset of Shedding for Participants Infected With SARS-CoV-2 Who Had at Least 2 Positive Swab Samples During Study Follow-up

Characteristic	Posterior mean values (95% CI)		
	Asymptomatic group (n = 24)	Mildly symptomatic group (n = 46)	Moderately or severely symptomatic group (n = 59)
Peak viral RNA load, Ct value	30.7 (29.8 to 31.4)	28.0 (27.3 to 28.5)	23.3 (22.6 to 24.0)
Time from shedding onset to peak viral RNA load, d	1.3 (1.0 to 1.6)	1.4 (1.2 to 1.9)	1.7 (1.2 to 2.2)
Time from peak viral RNA load to shedding cessation, d	10.4 (8.0 to 15.9)	10.5 (9.8 to 11.6)	10.3 (9.9 to 11.0)
Time from peak viral RNA load to mild symptom onset, d	NA	0.6 (0.4 to 0.9)	0.1 (−0.1 to 0.3)
Time from peak viral RNA load to moderate or severe symptom onset	NA	NA	2.1 (1.9 to 2.3)

Abbreviations: Ct, cycle threshold; NA, not applicable.

Among mildly symptomatic participants, symptom onset occurred, on average, 0.6 days (95% CI, 0.4-0.9 days) after peak viral load. Among the moderately or severely symptomatic group, the onset of mild symptoms was estimated to occur 0.1 days after peak viral load (95% CI, 0.1 days before to 0.3 days after peak). In comparison, moderate or severe symptoms generally occurred 2.1 days after peak viral load (95% CI, 1.9-2.3 days) (Table; Figure 3).

## Discussion

Our study provides a detailed description of the SARS-CoV-2 G614 viral trajectories assessed prospectively by viral RNA detection in a diverse, COVID-19 vaccine–naive, community-based outpatient cohort. The highest mean viral load occurred quickly after viral shedding onset and at a higher magnitude in patients with more severe COVID-19 symptoms. The viral shedding episodes had a sharp upswing to reach the peak viral load, followed by a prolonged decay. The duration of viral shedding varied widely and correlated with viral load peak. Our study complements and strengthens previously published observations and estimations of SARS-CoV-2 viral trajectories by providing a thorough evaluation with frequent molecular testing and detailed symptom reports collected daily after exposure to individuals with COVID-19.

Our study confirms data from previous studies that documented a variable duration of viral RNA shedding^11,26,27,28,29^ and expands those findings by providing early viral detection in asymptomatic persons with incident SARS-CoV-2 infection. One systematic review evaluating viral shedding data from 28 studies showed that the median duration of viral shedding was 18 days after onset of symptoms, with the longest period of viral RNA detection, 83 days, in the upper respiratory tract.^30^ Most studies (97%) included in that review were conducted among hospitalized patients, with samples obtained after COVID-19 symptom onset. However, in an out-of-hospital cohort of 303 quarantined patients with laboratory-confirmed SARS-CoV-2 infection in Korea, the median duration of viral RNA detection of 17 to 19 days was similar to that observed in hospitalized patients.^31^ Although the asymptomatic and symptomatic patients included in that study received frequent SARS-CoV-2 testing from upper and lower respiratory tract samples (days 8, 9, 15, and 16 of isolation with an option to extend testing up to day 19), the lack of early sampling after exposure to index cases could have resulted in missed early viral detection and inaccurate estimation of viral duration.

In our analysis of 97 adults with incident infection, the duration of SARS-CoV-2 G614 shedding in nasal swab samples lasted from 1 to 13 days and was limited by the 14-day follow-up. Almost half of the participants had a single positive swab sample with lower quantities of SARS-CoV-2 detected compared with participants with multiple positive swab samples. Although our study enrolled participants who reported exposure to at least 1 laboratory-confirmed case in their household or close contacts, precise exposures and the timing of transmissions are challenging to validate. Some participants may have had asymptomatic infections prior to enrollment, and we may have captured the end of their infection when viral detection could be intermittent.^12,15,32,33^ Single-day detection of SARS-CoV-2 RNA could also be associated with the size of the viral inoculum, allowing for innate immunity to clear the virus efficiently.^34^ Alternatively, immune priming by prior seasonal coronaviruses may have contributed to limited infections.^35,36,37^ Furthermore, a few participants may have had preexisting adaptive immunity from prior SARS-CoV-2 infection because we did not document their antibody status at enrollment.^38,39,40^ Because the duration of SARS-CoV-2 viral shedding is variable among individuals, frequent measurements with sensitive viral detection assays early after exposure are essential to quantify infection duration for fitting transmission models and for assessment of therapeutic efficacy in trials using viral load and infection duration as end points.

Our estimate of a rapid viral peak and subsequent slower decline has been demonstrated in prior mathematical models of SARS-CoV-2 infection and other respiratory viruses, such as respiratory syncytial virus, human rhinovirus, and H1N1, using molecular detection as well as chronic nonrespiratory viral infections, such as herpes simplex virus.^29,41,42,43,44,45^ Our observation of a lower viral load at the initiation of viral shedding with a peak viral load shortly after shedding onset suggests a rapid viral production after acquisition. The transition from the peak to viral decay occurs quickly, and the magnitude of the peak correlates with the duration of the shedding episode (intense and extended infection). The peak viral load differed according to COVID-19 clinical severity, with a higher estimated viral load in patients with greater symptom severity, suggesting that the copy number is associated with clinical disease. For participants reporting COVID-19 symptoms, moderate or severe symptoms started shortly after the peak viral load was reached, as demonstrated by others.^29^ Our results of higher viral load in symptomatic rather than asymptomatic persons agree with findings in a recent community-based study evaluating RNA levels in self-collected nasal swab samples from 550 adults and children in Washington state^46^ and a small prospective study of 31 patients hospitalized in China.^47^ However, other studies did not find associations between viral load and disease severity.^27,48^ Emerging data indicate that the SARS-CoV-2 viral load in respiratory samples is an important factor associated with SARS-CoV-2 transmission^49^; therefore, an accurate measurement of viral load is an important parameter to develop mathematical models of viral spread. This is particularly important because preliminary findings indicate that new variants of concern are associated with a significantly higher viral load than variants circulating early in the pandemic, potentially explaining their greater transmissibility.^50,51^

Acute respiratory viral infections pose multiple challenges in the evaluation of therapeutics. The poor specificity of clinical symptoms for particular pathogens, the variability in the magnitude and duration of viral shedding, and the short window of viral replication may hamper our the ability to evaluate candidate antiviral agents. Pragmatically, the SARS-CoV-2 viral dynamics also create challenges to devise scalable treatments that can be given early enough to affect the peak viral load and modify the natural history of the disease. With the widespread use of the COVID-19 vaccines, placebo-controlled clinical trials for evaluation of new preventive interventions will become more challenging to design and implement. These data provide an accurate assessment of viral trajectories in the absence of effective intervention and can form the basis for the design of future trials or essential historical controls.

### Limitations

This study has some limitations. The self-sampling technique or the degradation of the sample could yield false-negative results. However, the unsupervised collection of midturbinate swab samples for the dectection of SARS-CoV-2 has been demonstrated to be comparable to clinician-collected swab samples, and the samples are stable at ambient temperature for up to 9 days.^52,53^ The universal detection of ribonuclease P DNA confirmed that participants successfully collected valid samples, and the high incidence of SARS-CoV-2 based on self-collected swab samples suggests that self-sampling was adequate. Our analysis was limited to a 14-day observation period, which underestimates shedding duration owing to right censoring. Furthermore, despite collecting frequent swab samples early after exposure to persons with confirmed SARS-CoV-2 infection, daily swabbing could have missed the peak viral load in those with shorter viral shedding duration. Although SARS-CoV-2 RT-PCR is the preferred diagnostic test to confirm infection, we acknowledge that detectable viral RNA does not equal culture positivity or infectiousness. Furthermore, our study relied on Ct values as a proxy for viral RNA levels. Our viral shedding model assumed that viral load followed a piecewise linear trend, whereas the true trajectories might be more curved. Owing to incomplete observed trajectories, the model-based trajectories for some participants were associated with large uncertainties.

## Conclusions

Our cohort study of the early SARS-CoV-2 G614 variant infections suggests that viral replication peaks rapidly, followed by a slow decrease, suggesting that the period for effective antiviral intervention is very narrow. Our longitudinal evaluation of the SARS-CoV-2 G614 variant before implementation of COVID-19 vaccines may serve as a reference for comparison of emergent viral lineages to inform clinical trial design and public health strategies to contain the spread of the virus and to inform the development of ongoing therapeutics for SARS-CoV-2.

## Notes

Supplement. **eTable 1.** COVID-19 Symptoms Severity Definition **eTable 2.** Characteristics of Participants for Each Study Cohort **eTable 3.** Summary of Severe Acute Respiratory Syndrome Coronavirus-2 (SARS-CoV-2) Lineages Observed in the Study **eFigure 1.** Flow Chart Illustrating Participant Cohorts Included in the Study **eMethods.** Piecewise Linear Mixed-Effects Model for Viral RNA Load Trajectory Estimation **eFigure 2.** A Theoretical Severe Acute Respiratory Syndrome Coronavirus-2 (SARS-CoV-2) Viral RNA Load Trend (Blue Line) Overlaying the Observed Viral Load for a Participant in the Study **eFigure 3.** Distribution of the RNAse P Cycle Threshold (Ct) Values **eFigure 4.** The Observed Viral RNA Load Trajectories for the 97 Participants With Incident Severe Acute Respiratory Syndrome Coronavirus-2 (SARS-CoV-2) Infection **eFigure 5.** The Model Estimated Individual-Level Viral RNA Load Trajectories for the 129 Participants Who Had 2 or More Swabs Positive for Severe Acute Respiratory Syndrome Coronavirus-2 (SARS-CoV-2) RNA During the 14 Days of Follow-up in the Parent Study **eFigure 6.** Posterior Density Plots of Population-Level Severe Acute Respiratory Syndrome Coronavirus-2 (SARS-CoV-2) Viral RNA Load Trajectory Characteristics From the Fitted Models
eReferences.
