# Supplementary material for: Trajectory of Viral RNA Load Among Persons With Incident SARS-CoV-2 G614 Infection (Wuhan Strain) in Association With COVID-19 Symptom Onset and Severity
Source: JAMA Netw Open. 2022 Jan 10;5(1):e2142796. doi: 10.1001/jamanetworkopen.2021.42796 (PMC8749477; doi:10.1001/jamanetworkopen.2021.42796)
Supplement: Supplement. — eTable 1. COVID-19 Symptoms Severity Definition eTable 2. Characteristics of Participants for Each Study Cohort eTable 3. Summary of Severe Acute Respiratory Syndrome Coronavirus-2 (SARS-CoV-2) Lineages Observed in the Study eFigure 1. Flow Chart Illustrating Participant Cohorts Included in the Study eMethods. Piecewise Linear Mixed-Effects Model for Viral RNA Load Trajectory Estimation eFigure 2. A Theoretical Severe Acute Respiratory Syndrome Coronavirus-2 (SARS-CoV-2) Viral RNA Load Trend (Blue Line) Overlaying the Observed Viral Load for a Participant in the Study eFigure 3. Distribution of the RNAse P Cycle Threshold (Ct) Values eFigure 4. The Observed Viral RNA Load Trajectories for the 97 Participants With Incident Severe Acute Respiratory Syndrome Coronavirus-2 (SARS-CoV-2) Infection eFigure 5. The Model Estimated Individual-Level Viral RNA Load Trajectories for the 129 Participants Who Had 2 or More Swabs Positive for Severe Acute Respiratory Syndrome Coronavirus-2 (SARS-CoV-2) RNA During the 14 Days of Follow-up in the Parent Study eFigure 6. Posterior Density Plots of Population-Level Severe Acute Respiratory Syndrome Coronavirus-2 (SARS-CoV-2) Viral RNA Load Trajectory Characteristics From the Fitted Models eReferences. [file jamanetwopen-e2142796-s001.pdf]

# Supplementary Online Content

Stankiewicz Karita HC, Dong TQ, Johnston C, et al. Trajectory of viral RNA load among persons with incident SARS-CoV-2 G614 infection (Wuhan strain) in association with COVID-19 symptom onset and severity. *JAMA Netw Open.* 2022;5(1):e2142796. doi:10.1001/jamanetworkopen.2021.42796

**eTable 1.** COVID-19 Symptoms Severity Definition

**eTable 2.** Characteristics of Participants for Each Study Cohort

**eTable 3.** Summary of Severe Acute Respiratory Syndrome Coronavirus-2 (SARS-CoV-2) Lineages Observed in the Study

**eFigure 1.** Flow Chart Illustrating Participant Cohorts Included in the Study

**eMethods.** Piecewise Linear Mixed-Effects Model for Viral RNA Load Trajectory Estimation

**eFigure 2.** A Theoretical Severe Acute Respiratory Syndrome Coronavirus-2 (SARS-CoV-2) Viral RNA Load Trend (Blue Line) Overlaying the Observed Viral Load for a Participant in the Study

**eFigure 3.** Distribution of the RNAse P Cycle Threshold (Ct) Values

**eFigure 4.** The Observed Viral RNA Load Trajectories for the 97 Participants With Incident Severe Acute respiratory Syndrome Coronavirus-2 (SARS-CoV-2) Infection

**eFigure 5.** The Model Estimated Individual-Level Viral RNA Load Trajectories for the 129 Participants Who Had 2 or More Swabs Positive for Severe Acute Respiratory Syndrome Coronavirus-2 (SARS-CoV-2) RNA During the 14 Days of Follow-up in the Parent Study

**eFigure 6.** Posterior Density Plots of Population-Level Severe Acute Respiratory Syndrome Coronavirus-2 (SARS-CoV-2) Viral RNA Load Trajectory Characteristics From the Fitted Models

**eReferences.**

This supplementary material has been provided by the authors to give readers additional information about their work.

© 2022 Stankiewicz Karita HC et al. *JAMA Network Open.*

**eTable 1.** COVID-19 Symptoms Severity Definition

COVID-19 symptoms severity levels were defined in reference to the Centers for Disease Control and Prevention (CDC) clinical criteria.[1] A person was mildly/moderately/severely symptomatic if he/she reached mild/moderate/severe levels in at least two of the following symptoms: fever chills, myalgia, headache, sore throat, nausea or vomiting, diarrhea, fatigue, congestion or runny nose; or any one of the following symptoms: cough, shortness of breath, new olfactory disorder, new taste disorder.

| Symptom | | Categorization of symptom severity provided by participants using daily REDCap surveys* | | |
|---|---|---|---|---|
| | | Mild | Moderate | Severe |
| Category 1 | Fever (measured or subjective) | fever = 1 | fever = 2 | fever ≥ 3 |
| | Chills | chills = 1 | chills = 2 | chills ≥ 3 |
| | Myalgia | muscle aches = 1 | muscle aches = 2 | muscle aches ≥ 3 |
| | Headache | headache = 1 | headache = 2 | headache ≥ 3 |
| | Sore throat | sore throat = 1 | sore throat = 2 | sore throat ≥ 3 |
| | Nausea or vomiting | nausea = 1 OR vomiting = 1 | nausea = 2 OR vomiting = 2 | nausea ≥ 3 OR vomiting ≥ 3 |
| | Diarrhea | diarrhea = 1 | diarrhea = 2 | diarrhea ≥ 3 |
| | Fatigue | fatigue = 1 | fatigue = 2 | fatigue ≥ 3 |
| | Congestion or runny nose | nasal congestion = 1 OR runny nose = 1 | nasal congestion = 2 OR runny nose = 2 | nasal congestion ≥ 3 OR runny nose ≥ 3 |
| Category 2 | Cough | dry cough = 1 | dry cough = 2 | dry cough ≥ 3 |
| | Shortness of breath | shortness of breath = 1 | shortness of breath = 2 | shortness of breath ≥ 3 |
| | New olfactory disorder | changes to smell = 1 | changes to smell = 2 | changes to smell ≥ 3 |
| | New taste disorder | changes to taste = 1 | changes to taste = 2 | changes to taste ≥ 3 |
| Overall | COVID-19 symptom | [At least two of the category 1 symptoms are mild or more severe<br>OR<br>At least one of the category 2 symptoms are mild or more severe]<br>AND<br>[Overall COVID-19 symptom is NOT moderate or severe]. | [At least two of the category 1 symptoms are moderate or more severe<br>OR<br>At least one of the category 2 symptoms are moderate or more severe]<br>AND<br>[Overall COVID-19 symptom is NOT severe]. | [At least two of the category 1 symptoms are severe<br>OR<br>At least one of the category 2 symptoms are severe] |
| *Participants were asked to rate each symptom on a 5-point ordinal scale: 0 ("none"), 1 ("mild - does not interfere with daily activities"), 2 ("moderate – interferes with daily activity"), 3 ("severe – prevents daily activities"), 4 ("Emergency room or hospitalization").[2] | | | | |

© 2022 Stankiewicz Karita HC et al. *JAMA Network Open.*

**eTable 2.** Characteristics of Participants for Each Study Cohort

| Variables | Participants with incident SRAS-CoV-2 infection (N = 97) | Participants with ≥2 positive swabs during follow-up (N = 129) |
|---|---|---|
| Median age ($Q_1$, $Q_3$), y | 37 (27, 52) | 38 (25, 54) |
| Sex — no. (%) | | |
| Women | 55 (57) | 58 (53) |
| Men | 42 (43) | 60 (47) |
| Other | 0 (0) | 1 (1) |
| Race and ethnic group — no. (%) | | |
| Asian | 17 (18) | 16 (12) |
| Black or African American | 10 (10) | 15 (12) |
| White | 53 (54) | 76 (59) |
| American Indian/Alaskan Native | 2 (2) | 4 (3) |
| Native Hawaiian or other Pacific Islander | 0 (0) | 1 (1) |
| Other/Prefer not to say | 15 (15) | 17 (13) |
| Hispanic or Latino ethnic group — no. (%) | 29 (30) | 39 (30) |
| Coexisting conditions — no. (%) | | |
| Metabolic diseases | 24 (25) | 29 (23) |
| Hypertension | 19 (19) | 25 (19) |
| Diabetes mellitus type 2 | 10 (10) | 10 (8) |
| Immunosuppressive diseases | 5 (5) | 11 (9) |
| Lung diseases (all asthma) | 10 (10) | 13 (10) |
| Contact type — no. (%) | | |
| Household/social contact | 85 (88) | 121 (94) |
| Healthcare worker | 12 (12) | 8 (6) |
| Median time ($Q_1$, $Q_3$), hr. | | |
| Total hours of contact with all potential index case(s)[a] in the past 96 hours | 24 (5, 72) | 28 (6, 64) |
| Time from last contact with potential index case(s) to first dose/survey | 43 (21, 74) | 42 (12, 72) |
| Number of contacts in same household enrolled in the study — no. (%) | | |
| 1 | 71 (73) | 83 (64) |
| 2 – 3 | 17 (18) | 29 (22) |
| 4 or more | 9 (9) | 17 (13) |

[a]Index case(s) is a person with a laboratory-confirmed diagnosis of SARS-CoV-2 infection. All participants in this analysis were exposed to at least one lab-confirmed index case but could be exposed to more persons with presumptive diagnosis of SARS-CoV-2 infection by CDC criteria (meets clinical and epidemiologic evidence with no confirmatory laboratory testing performed for COVID-19).

Abbreviations: years, y; hours, hr; first quantile, $Q_1$; third quantile, $Q_3$; coronavirus disease 2019, COVID-19.

© 2022 Stankiewicz Karita HC et al. *JAMA Network Open.*

**eTable 3.** Summary of Severe Acute Respiratory Syndrome Coronavirus-2 (SARS-CoV-2) Lineages Observed in the Study

A total of 535 samples with Ct<34, corresponding to 108 participants were sequenced. Sequencing libraries were prepared using the Swift Biosciences' Normalase amplicon panel and sequenced on Illumina Nextseq instruments using 2x150 reads. Genomes were assembled using a custom pipeline described previously.[3] Genomes with <10% Ns were selected for further analysis including clade assignment which was performed using the Pangolin (https://pangolin.cog-uk.io/, 10.1038/s41564-020-0770-5) and Nextclade (https://clades.nextstrain.org/) tools.

| Lineage | Number of samples |
|---|---|
| B.1 | 197 |
| B.1.1.291 | 49 |
| B.1.516 | 48 |
| B.1.369 | 47 |
| B.1.594 | 26 |
| B.1.371 | 21 |
| B.1.1 | 20 |
| B.1.240 | 19 |
| B.1.111 | 18 |
| B.1.509 | 15 |
| B.1.2 | 10 |
| B.1.108 | 9 |
| B.1.1.135 | 8 |
| B.1.1.434 | 8 |
| B.1.319 | 7 |
| B.1.495 | 7 |
| B.1.493 | 6 |
| B.1.1.186 | 5 |
| B.1.1.370 | 3 |
| B.1.1.231 | 2 |
| B.1.1.29 | 1 |
| B.1.1.306 | 1 |
| B.1.263 | 1 |
| B.1.36.36 | 1 |
| B.1.400 | 1 |
| B.1.503 | 1 |
| B.1.564 | 1 |
| B.1.576 | 1 |
| B.1.577 | 1 |
| B.1.605 | 1 |

© 2022 Stankiewicz Karita HC et al. *JAMA Network Open.*

**eFigure 1.** Flow Chart Illustrating Participant Cohorts Included in the Study

Cohort 1 (blue) includes 97 participants who had a negative swab at baseline and at least 1 poisitive swab in the subsequent days of follow-up. Cohort 2 (orange) includes 129 participants who had at least 2 posisitive swabs for SARS-CoV-2 during follow-up. Note that there is overlap between the two study cohorts. There are a total of 173 unique participants in these two study cohorts.

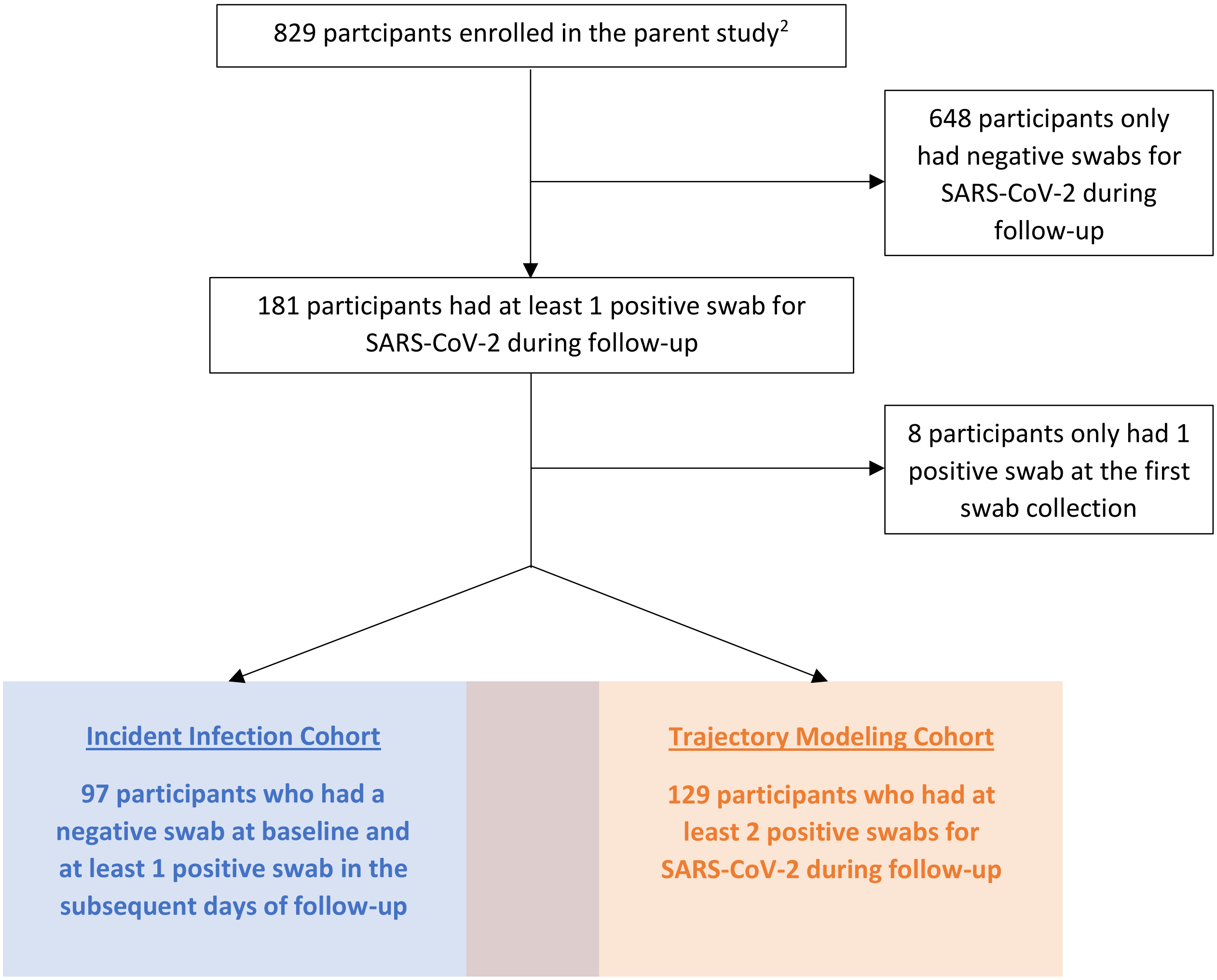


© 2022 Stankiewicz Karita HC et al. *JAMA Network Open.*

**eMethods.** Piecewise Linear Mixed-Effects Model for Viral RNA Load Trajectory Estimation

Since we only collected nasal swabs from each participant for 14 consecutive days, many (108 out of 180) participants who were infected with severe acute respiratory syndrome coronavirus-2 (SARS-CoV-2) during the follow-up period had censored shedding, i.e., their first and/or last collected swabs tested positive for SARS-CoV-2. We developed a Bayesian piece-wise linear mixed-effect model to estimate individual-level viral load trajectories and population-level viral load trajectory charactersitcs, including peak viral load, time from shedding onset to peak and time from peak to shedding cessation. We also summarized population-level timing of symptom onset in relation to peak viral load based on the model-estimated viral RNA load trajectories and the observed symptom onset times. The analysis was stratigied by participants' reported COVID-19 symptom severity.

## Data

We conducted model-based analysis using data from 129 participants who had 2 or more positive swabs during the 14 days of follow-up in the parent study to maximize data utilization from participants with sustained viral detection. The participants who only had 1 observed day of shedding were excluded from this model because we were unable to ascertain whether the single positive swab represented the tail of an infection acquired before study entry, a re-infection, or a false-positive result. The participants who were SARS-CoV-2 positive at baseline were included to enrich the observed viral load trajectory data, especially for estimating the rate of decay of viral load.

We divide the 129 participants into three symptom severity groups according to their reported COVID-19 symptoms (**eTable1**): (1) "asymptomatic" if no COVID-19 symptoms throughout follow-up; (2) "mildly symptomatic" if only reported mild COVID-19 symptoms during follow-up; (3) "moderately or severely symptomatic" if reported at least one day of moderate or severe COVID-19 symptoms during follow-up.

## Model description

We fitted a piece-wise linear mixed-effect model within each symptom severity group to estimate the peak viral load measured in cycle threshold (Ct) value, the time from shedding onset to peak viral load, and the time from peak to viral clearance. We assumed that the viral load trajectories follow a trend that consists of a proliferation phase with linear growth of viral load on the Ct scale, followed by a clearance phase with linear decay of viral load on the Ct scale. This corresponds to exponential growth and decay in viral RNA concentration in the respective phases. This idealized trajectory is depicted in **eFigure2** of this supplement and represented by the following equations:

$$E[Ct(s)] = 40 - \mu_y(s)$$

$$\mu_{y(s)} = v_p \times I[-w_a \le s \le w_b] + \frac{v_p}{w_a} \times s \times I[-w_a \le s \le w_b] - \frac{v_p}{w_b} \times s \times I[-w_a \le s \le w_b]$$

Here, $I(\cdot)$ represents an indicator function. $s$ represents time since the peak viral load of the idealized viral trajectory, so that $s = 0$ at the peak of the trajectory. $E[Ct(s)]$ represents the expected value of viral load in Ct at time $s$. $\mu_y(s)$ represents the difference between the level of detection (LOD, equals 40) and the expected viral load at time $s$. $v_p$ represents the absolute difference between LOD and the peak viral load in Ct. $w_a$represents time in days between shedding onset and peak viral load, and $w_b$represents time in days between peak viral load and viral clearance. $t_p$ represents the time difference between the latent peak viral load and the observed peak viral load.

We assumed that the difference between the observed viral load and the LOD, $y(s)$, has the following distribution:

$$y(s) \sim \text{Normal}\left(\mu_y(s), \sigma_y(s)\right)$$

$$\sigma_y(s) = \sigma_{yy} \times I[-w_a \le s \le w_b] + 0.1 \times (1 - I[-w_a \le s \le w_b])$$

© 2022 Stankiewicz Karita HC et al. *JAMA Network Open.*

This model assumes that the observed Ct values are normally distributed around the expected trajectory with standard error $\sigma_{yy}$ during the viral shedding, and with standard error $0.05$ before and after viral shedding to allow for deviation from LOD due to potential errors from various sources such as misplaced swabs.
We used random effects to capture individual-level variations from the population-level means (μ) and specified the following distributions for the random effects:

$$t_{p,i} \sim_{i.i.d.} \text{Normal}(\mu_{tp}, \sigma_{tp})$$

$$log([v_{p,i}, w_{a,i}, w_{b,i}]) \sim \text{Multivariate Normal}(\mu_{log}, \Sigma_{log})$$

Here, $\mu_{log}$ is a vector of length 3 and $\Sigma_{log}$ is a 3 by 3 variance-covariance matrix.
We used a Markov Chain Monte Carlo (MCMC) fitting procedure implemented in JAGS (version 4.3.0)[4] and R (version 4.1.1)[5] to estimate the individual-level and population-level parameters. We incorporated information from previous work[6] by specifying the following prior distributions for the population-level means:

$$\mu_{tp} \sim \text{Normal}(0,1)$$

$$\mu_{log}[1] \sim \text{Normal}(\log(18), 2), \text{truncated}[-\infty, \log(40)]$$

$$\mu_{log}[2] \sim \text{Normal}(log(3), 1)$$

$$\mu_{log}[3] \sim \text{Normal}(log(9), 1)$$

We used weakly-informative priors for the rest of the parameters:

$$\sigma_{tp} \sim \text{Cauchy}(0,1), \text{truncated}[0, \infty]$$

$$\sigma_{yy} \sim \text{Cauchy}(0,1), \text{truncated}[0, \infty]$$

$$\Sigma_{log} \sim \text{Inverse Wishart}(I_{3\times3}, 4)$$

We ran an MCMC chain for 10,000 iterations, with the first 5,000 iterations discarded as burn-in. Using a thinning interval of 5, we used 1,000 of the second 5,000 iterations for inference.

**Summary results**

The posterior distributions of key population-level parameters are shown in **eFigure6**. Specifically, we created the density plots of 1,000 posterior samples of the population-level mean (a) peak viral load, 40 - $v_p$, (b) time from shedding onset to peak viral load, $w_a$,(c) time from peak viral load to viral clearance, $w_b$, and (d) total duration of shedding, $w_a + w_b$. Specifically, for each quantity, we first obtain the average value across individuals in each posterior iteration. We then plot the density of the average values across the 1000 posterior iterations.

To estimate the population-level time from peak viral RNA load to mild symptom onset among the mildly symptomatic participants, we use the following procedure: First, for each posterior iteration and each individual, we obtained the latent time of peak viral load. Second, for each posterior iteration and individual, we obtained the time from the latent peak to the observed mild symptom onset. Next, for each posterior iteration, we calculated the average time from the latent peak to the observed mild symptom onset across individuals. Last, we summarized the distribution of the average time across 1000 posterior iterations. Similar procedures were used to estimate the population-level time from peak viral RNA load to mild and moderate or severe symptom onset among the moderately or severely symptomatic participants.

© 2022 Stankiewicz Karita HC et al. *JAMA Network Open.*

**eFigure 2.** A Theoretical Severe Acute Respiratory Syndrome Coronavirus-2 (SARS-CoV-2) Viral RNA Load Trend (Blue Line) Overlaying the Observed Viral Load for a Participant in the Study

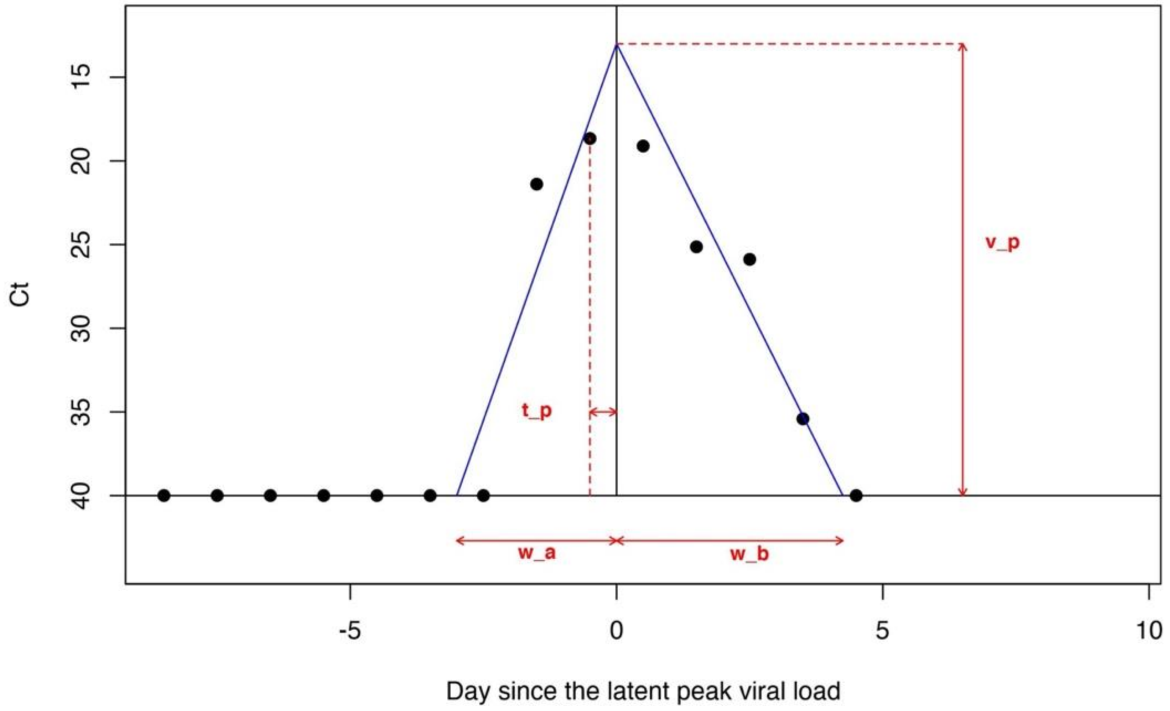


© 2022 Stankiewicz Karita HC et al. *JAMA Network Open.*

**eFigure 3.** Distribution of the RNAse P Cycle Threshold (Ct) Values

(a) Distribution of the RNAse P Ct values of all swabs with RNase P detected, grouped by the severe acute respiratory syndrome coronavirus-2 (SARS-CoV-2) PCR status. Each dot represents one observed RNAse P Ct value obtained among all study participants in the parent study.[2] The horizontal line segments are the median RNAse P Ct values categorized by SARS-CoV-2 detection. A total of 1207 swabs were tested for RNAse P, 1126 were SARS-CoV-2 negative, 81 were SARS-CoV-2 positive. A total of 1197 (99%) swabs had RNAse P detected, 1116 were SARS-CoV-2 negative, and 81 were SARS-CoV-2 positive.

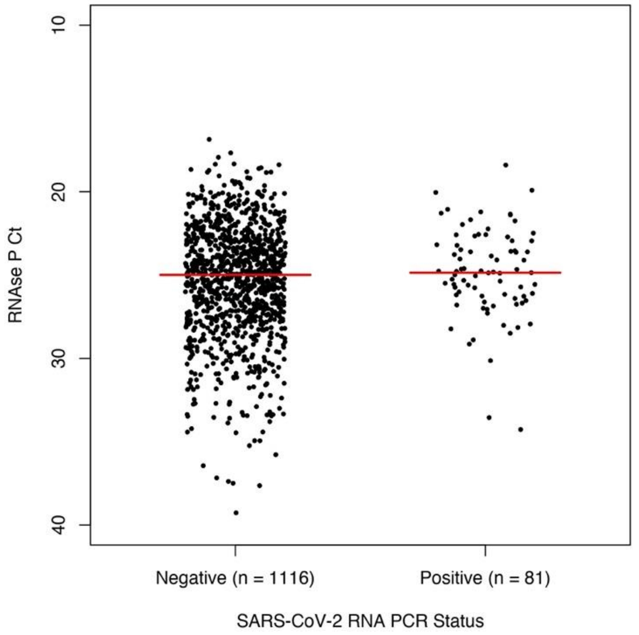


(b) Scatterplot of the RNAse P Ct values against the SARS-CoV-2 viral load Ct values among all positive SARS-CoV-2 samples that were tested for RNAse P in the parent study (n = 81), with the locally weighted scatterplot smoothing (LOWESS) curve and the 95% confidence interval.

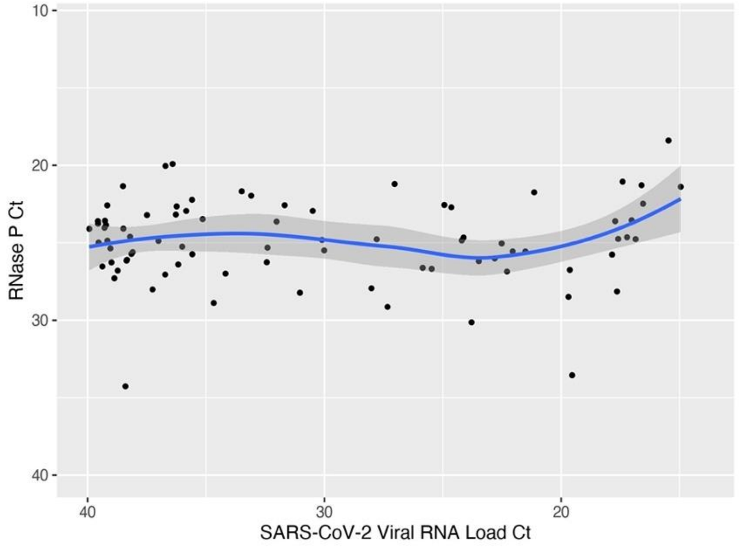


© 2022 Stankiewicz Karita HC et al. *JAMA Network Open.*

**eFigure 4.** The Observed Viral RNA Load Trajectories for the 97 Participants With Incident Severe Acute Respiratory Syndrome Coronavirus-2 (SARS-CoV-2) Infection

Each "PID" represents a participant and the number of days in parentheses indicates the observed duration of SARS-CoV-2 shedding, with "N+" days indicating censoring. Each dot represents one self-collected mid-turbinate swab with the cycle threshold (Ct) value represented on the y-axis. The color indicates the reported COVID-19 symptom severity: "mild" if symptoms did not interfere with daily activities; "moderate" if symptoms interfered with daily activities; and "severe" if symptoms prevented daily activities, require an emergency room visit and/or hospitalization.

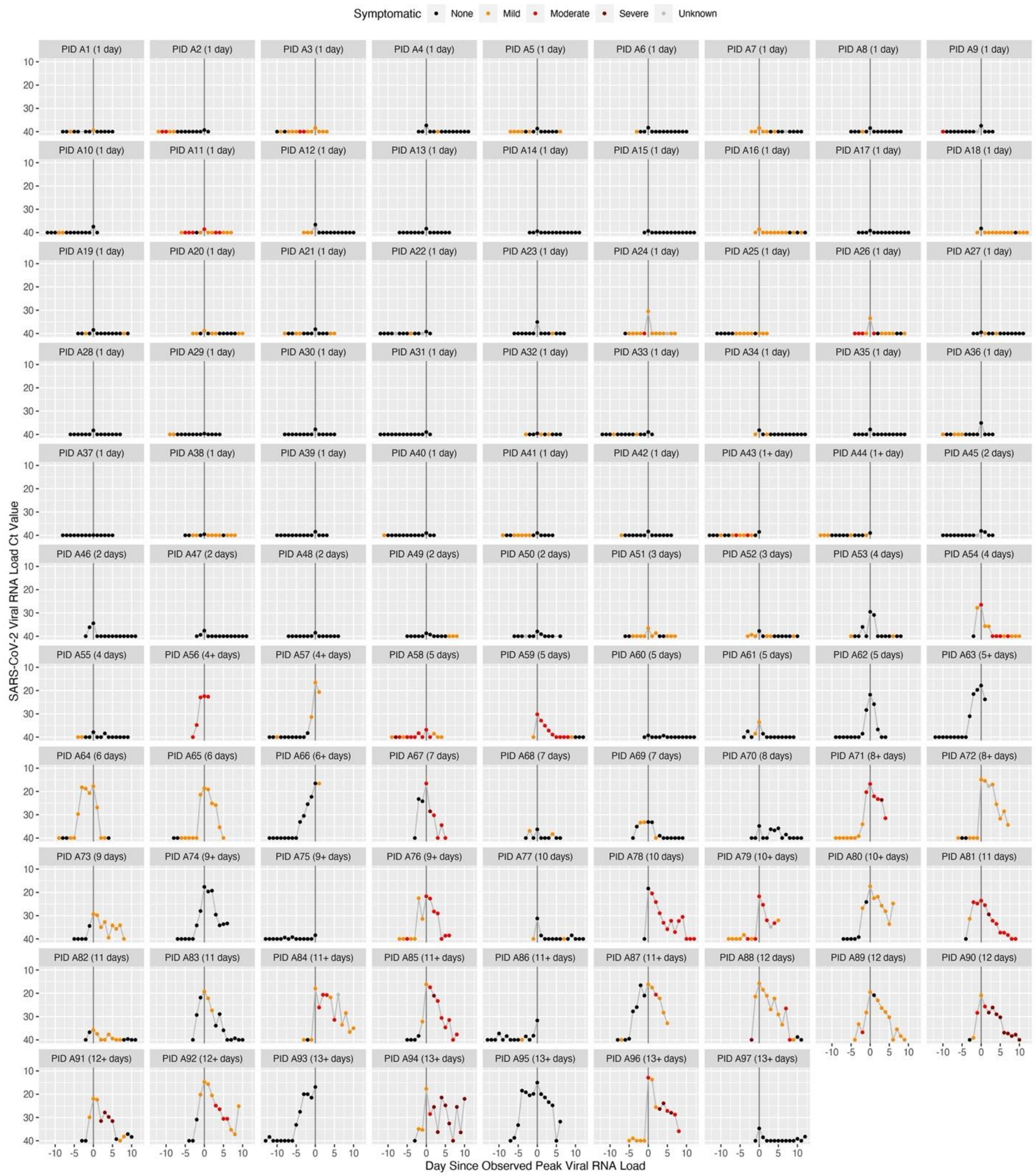


© 2022 Stankiewicz Karita HC et al. *JAMA Network Open.*

**eFigure 5.** The Model Estimated Individual-Level Viral RNA Load Trajectories for the 129 Participants Who Had 2 or More Swabs Positive for Severe Acute Respiratory Syndrome Coronavirus-2 (SARS-CoV-2) RNA During the 14 Days of Follow-up in the Parent Study

The x-axis was centered at the day of the observed peak viral load. The lines and ribbons were the posterior mean and the 90% credible interval of SARS-CoV-2 viral shedding, and the colored dots were the observed SARS-CoV-2 viral cycle threshold (Ct) values from Polymerase Chain Reaction (PCR) tests, colored by symptom severity.

(a) Model estimated individual-level viral RNA load trajectories for people in the asymptomatic group.

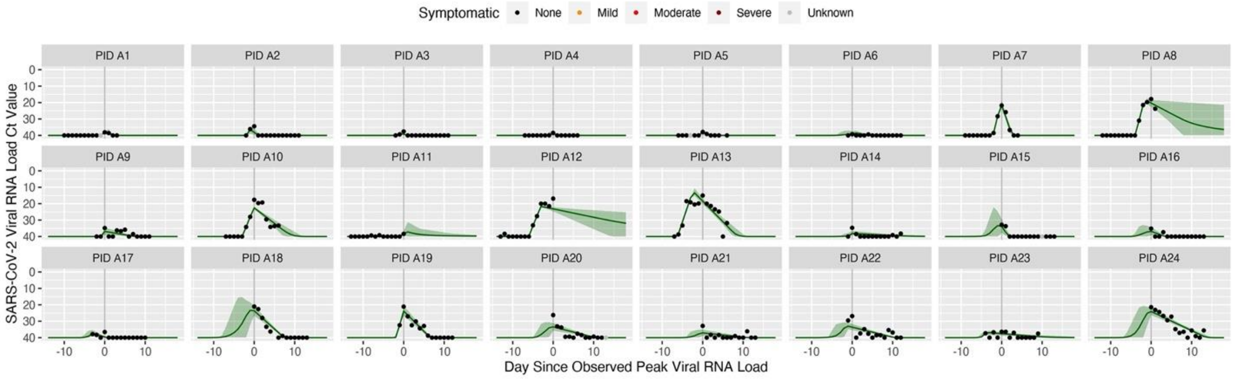


(b) Model estimated individual-level viral RNA load trajectories for people in the mildly symptomatic group

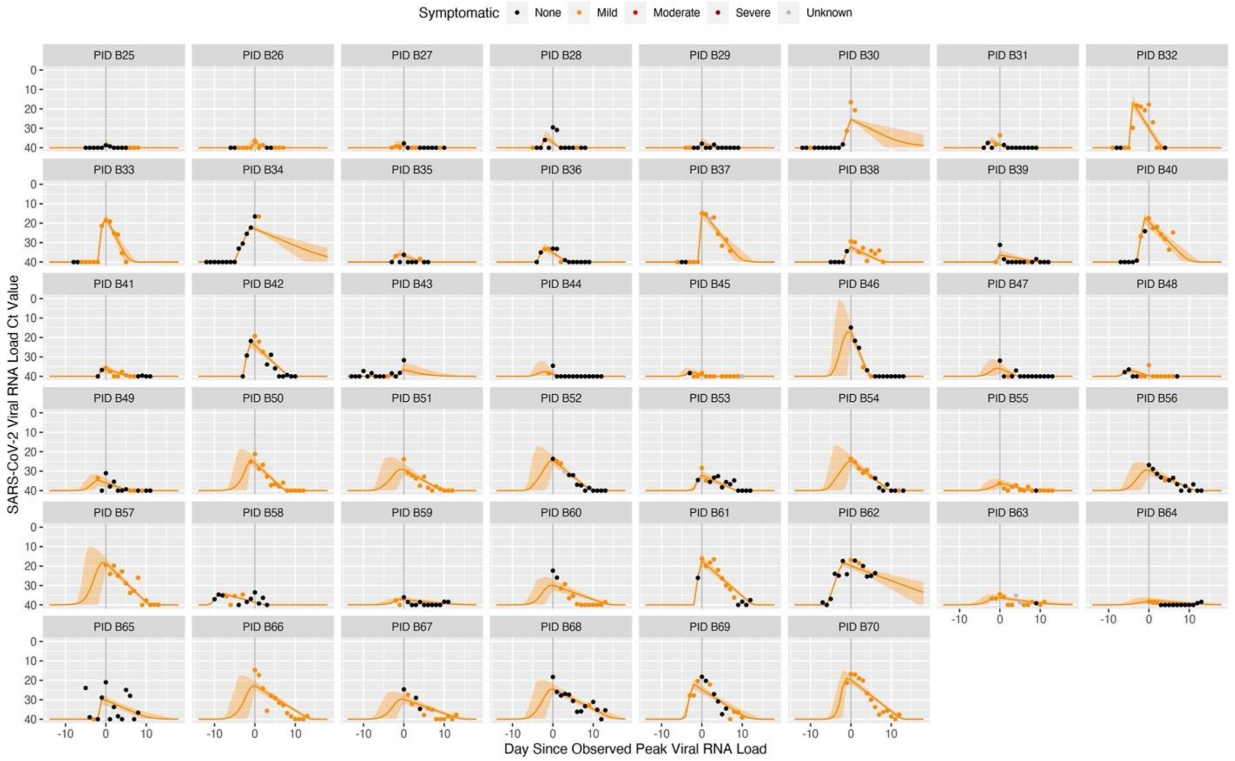


© 2022 Stankiewicz Karita HC et al. *JAMA Network Open.*

(c) Model estimated individual-level viral RNA load trajectories for people in the moderately/severely symptomatic group.

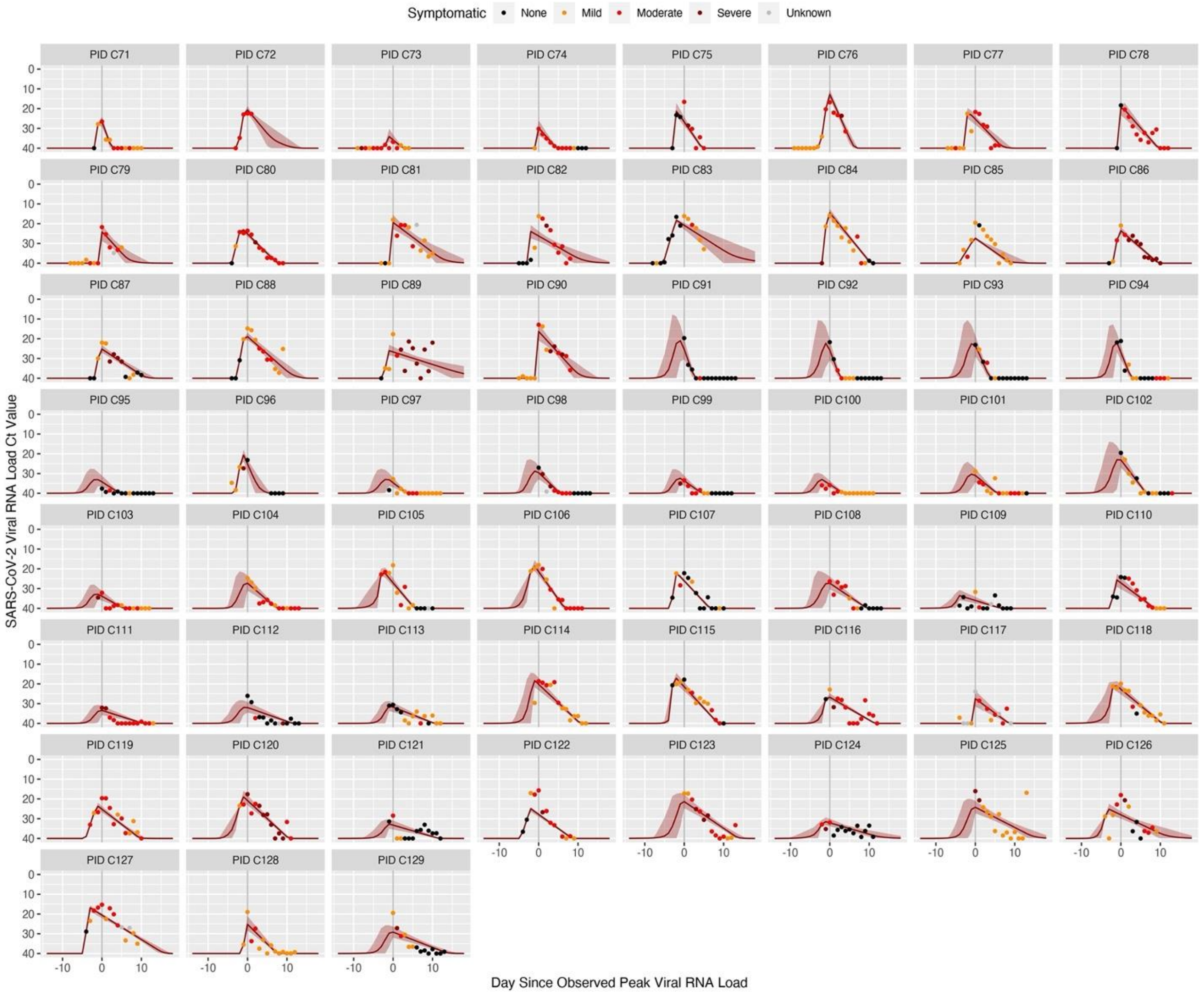


© 2022 Stankiewicz Karita HC et al. *JAMA Network Open*.

**eFigure 6.** Posterior Density Plots of Population-Level Severe Acute Respiratory Syndrome Coronavirus-2 (SARS-CoV-2) Viral RNA Load Trajectory Characteristics From the Fitted Models

(a) Posterior density plot of the population-level peak viral RNA load by symptom groups.

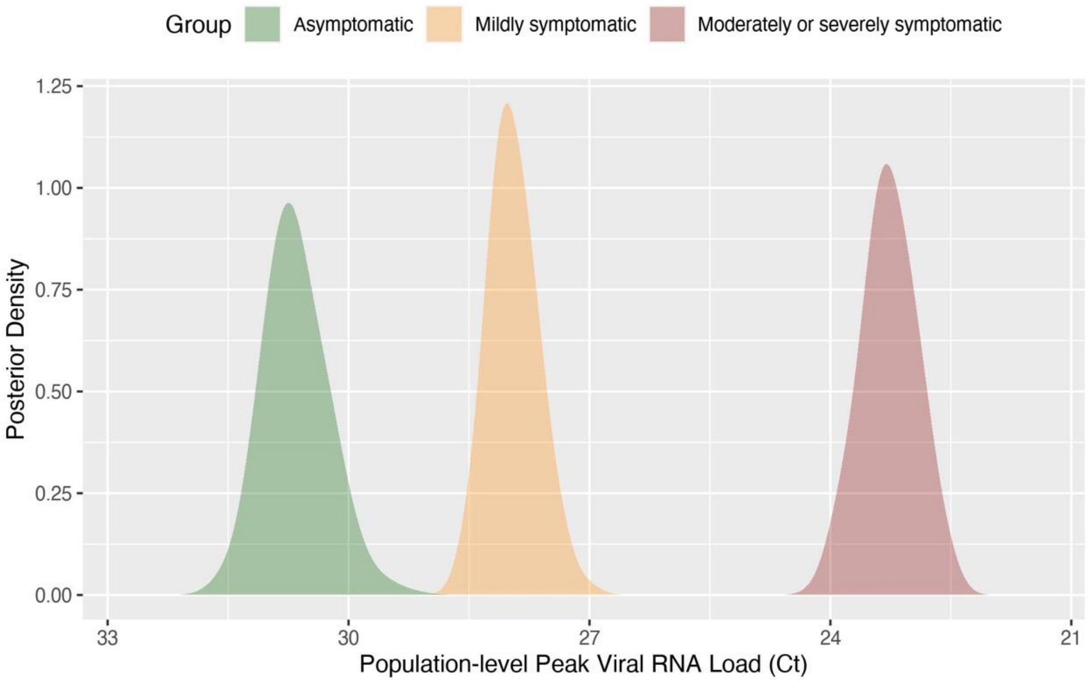


(b) Posterior density plot of the population-level time from shedding onset to peak viral RNA load by symptom groups.

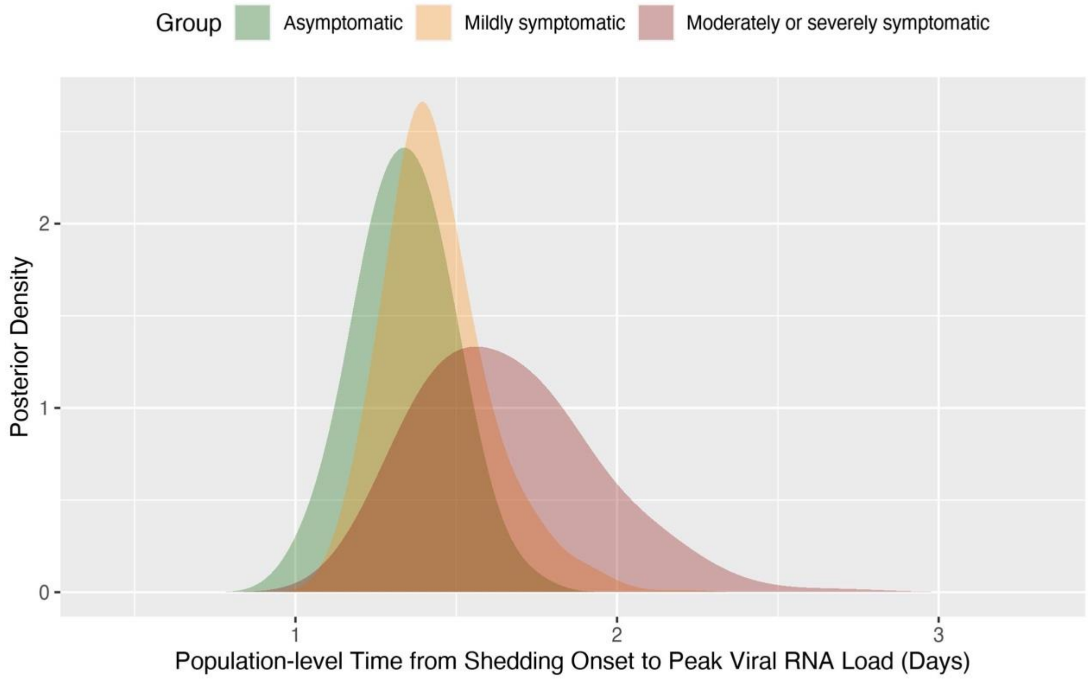


© 2022 Stankiewicz Karita HC et al. *JAMA Network Open.*

(c) Posterior density plot of the population-level time from peak viral RNA load to shedding cessation by symptom groups.

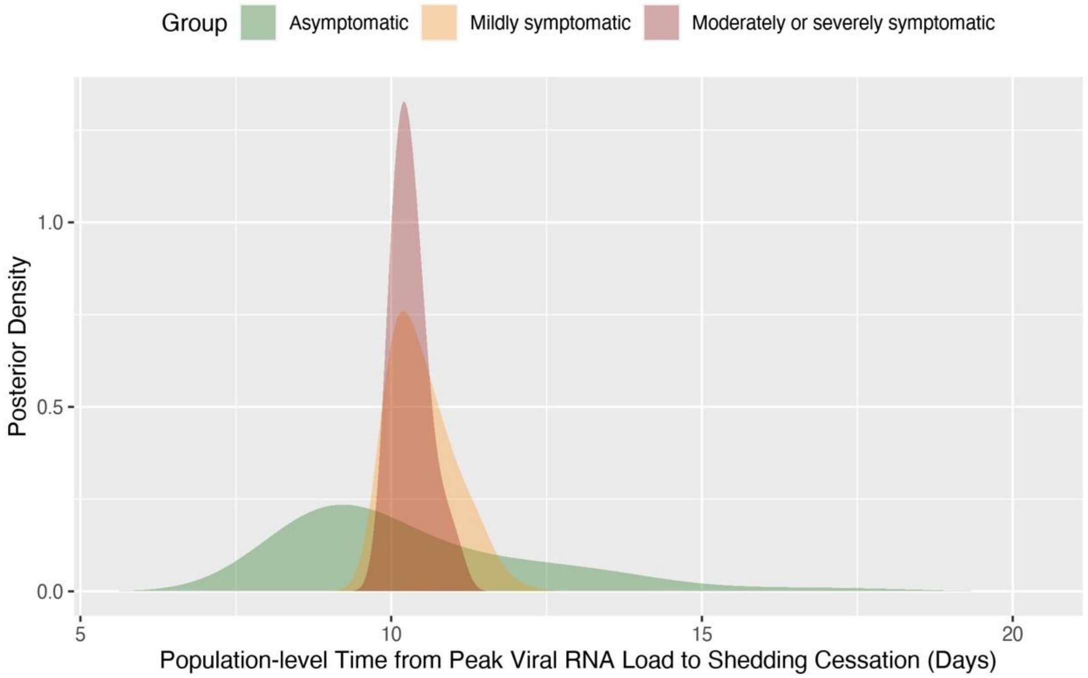


(d) Posterior density plot of the population-level total duration of shedding by symptom groups.

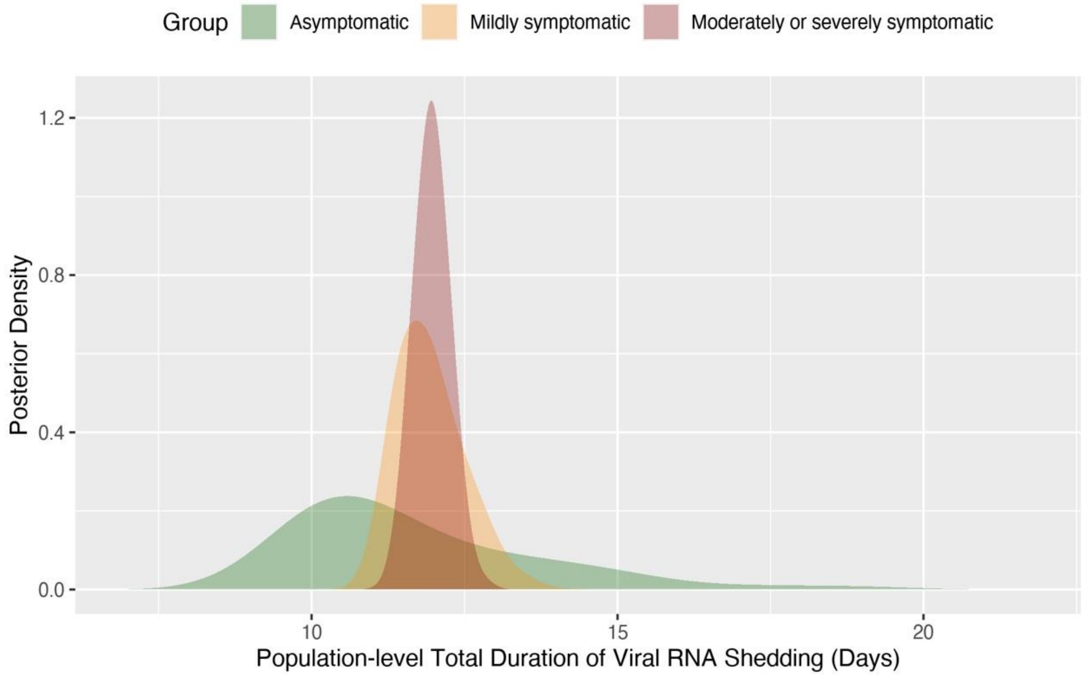


© 2022 Stankiewicz Karita HC et al. *JAMA Network Open.*

(e) Posterior density plot of the population-level time from peak viral RNA load to mild symptom onset by symptom groups.

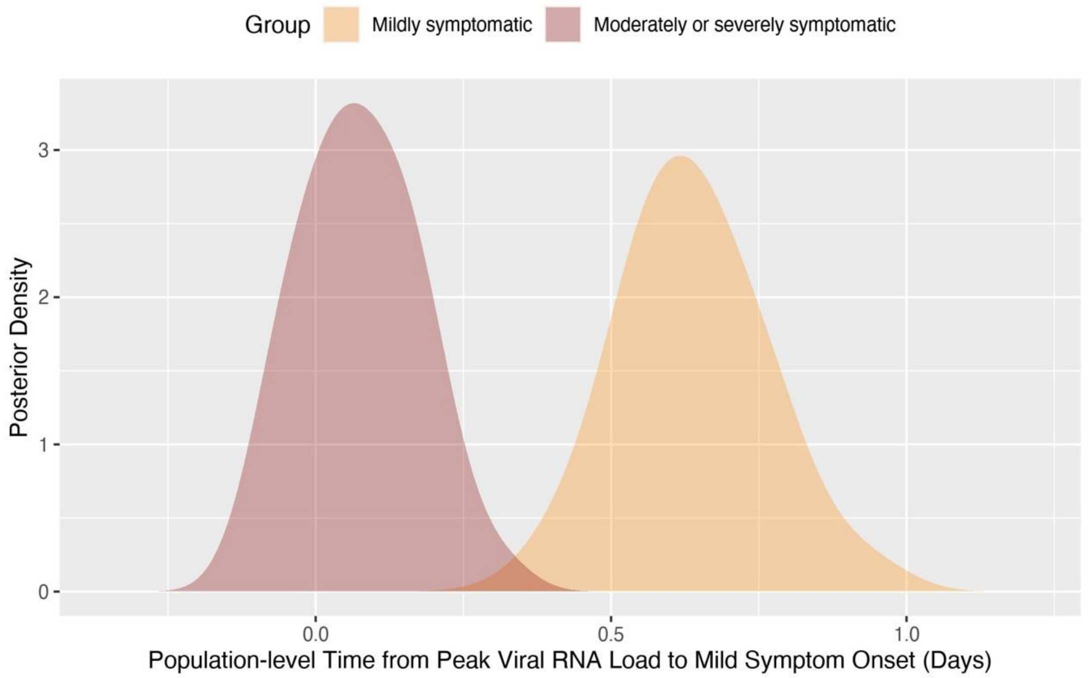


(f) Posterior density plot of the population-level time from peak viral RNA load to moderate or severe symptom onset.

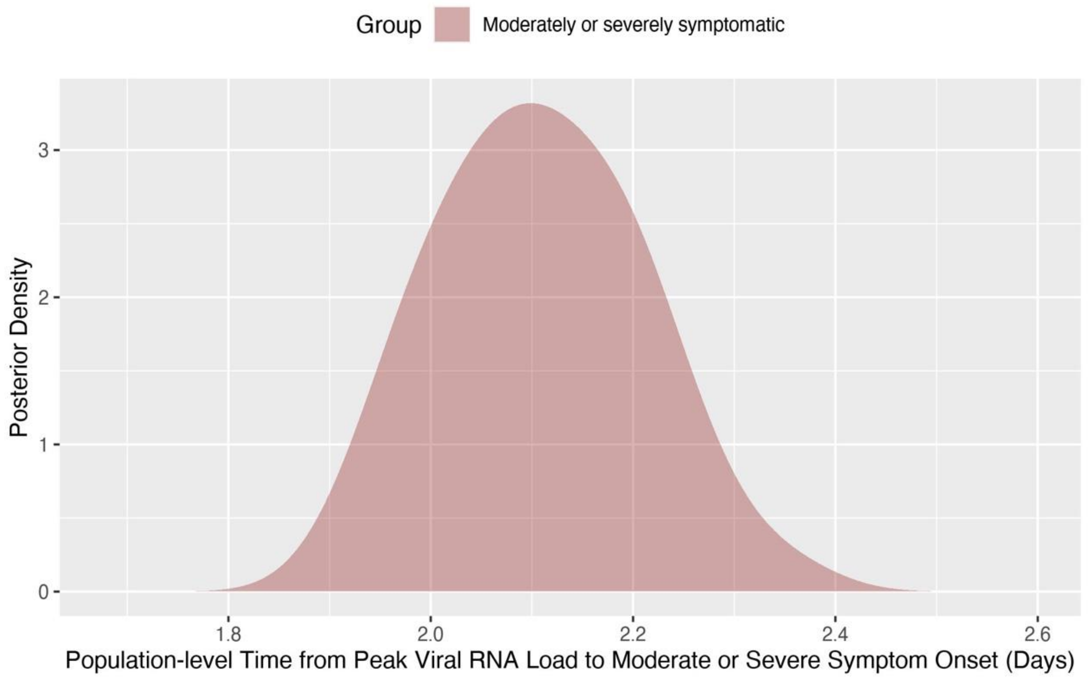


© 2022 Stankiewicz Karita HC et al. *JAMA Network Open.*

## eReferences.


1. Coronavirus Disease 2019 (COVID-19) | CDC. Available at: https://ndc.services.cdc.gov/case-definitions/coronavirus-disease-2019-2020-08-05/.

2. Barnabas R V., Brown ER, Bershteyn A, et al. Hydroxychloroquine as Postexposure Prophylaxis to Prevent Severe Acute Respiratory Syndrome Coronavirus 2 Infection: A Randomized Trial. Ann Intern Med. 2021;174(3):344-352.

3. Addetia A, Lin MJ, Peddu V, Roychoudhury P, Jerome KR, Greninger AL. Sensitive Recovery of Complete SARS-CoV-2 Genomes from Clinical Samples by Use of Swift Biosciences' SARS-CoV-2 Multiplex Amplicon Sequencing Panel. J Clin Microbiol. 2020;59(1):e02226-20.

4. Plummer M. JAGS: A program for analysis of Bayesian graphical models using Gibbs sampling. *Proceedings of the 3rd International Workshop on Distributed Statistical Computing. 2003;124.*

5. R Core Team (2021). R: A Language and Environment for Statistical Computing. *R Foundation for Statistical Computing*. 2021.

6. Kissler SM, Fauver JR, Mack C, et al. Viral dynamics of acute SARS-CoV-2 infection and applications to diagnostic and public health strategies. *PLoS Biology*. 2021;19(7).


© 2022 Stankiewicz Karita HC et al. *JAMA Network Open.*
